# Effect of Resveratrol-Enriched Rice on Skin Inflammation and Pruritus in the NC/Nga Mouse Model of Atopic Dermatitis

**DOI:** 10.3390/ijms20061428

**Published:** 2019-03-21

**Authors:** Min Cheol Kang, Kyohee Cho, Jae Hyuk Lee, Lalita Subedi, Silvia Yumnam, Sun Yeou Kim

**Affiliations:** 1 College of Pharmacy, Gachon University, 191, Hambakmoero, Yeonsu-gu, Incheon 21936, Korea; mincjf07@gmail.com (M.C.K.); kcho3138@gmail.com (K.C.); wogur6378@naver.com (J.H.L.); subedilali@gmail.com (L.S.); 2 Gachon Institute of Pharmaceutical Science, Gachon University, Yeonsu-gu, Incheon 21565, Korea

**Keywords:** atopic dermatitis, resveratrol-enriched rice, NC/Nga, dinitrochlorobenzene, skin inflammation

## Abstract

Resveratrol-enriched rice (RR) was developed using genetic engineering to combine the properties of resveratrol and rice. To evaluate the effect of RR on pruritic skin inflammation in atopic dermatitis (AD)-like skin lesions, we used dinitrochlorobenzene (DNCB)-induced NC/Nga mice and an in vitro 3D skin model. Normal rice (NR), resveratrol, and RR were topically applied to mice dorsal skin, following which the dermatitis index and scratching frequency were calculated. Histological examination was performed by hematoxylin and eosin and immunohistochemistry staining of IL-31 level. The level of immunoglobulin E (IgE) and IL-31 in the serum was determined by enzyme-linked immunosorbent assay (ELISA). The cytotoxicity of RR and the expression levels of pro-inflammatory cytokines were also determined in cultured human keratinocytes and a 3D skin model. RR significantly reduced scratching frequency, decreased the dermatitis severity and trans-epidermal water loss (TEWL) and improved skin hydration in DNCB-induced NC/Nga mice. RR also significantly decreased serum IL-31 and IgE levels and suppressed the production of IL-6 in human keratinocytes and the 3D skin model. Our study indicates that the synergistic effect of rice and resveratrol manifested by the topical application of RR can serve as a potential alternative therapy for chronic skin inflammatory diseases such as AD.

## 1. Introduction

Atopic dermatitis (AD) is a chronic inflammatory skin disease that has been characterized by pruritic eczematous lesions and skin barrier dysfunction [1]. AD affects up to 20% of children and 10% of adults in developed countries [2]. It develops from a complex interplay between genetic, environmental, and immunologic factors, which leads to multiple changes in the immune system [3]. The pathogenesis of AD is not yet fully understood, but the development of AD lesions is known to be associated with skin barrier dysfunction, cell-mediated immune response, and immunoglobulin E (IgE)-related hypersensitivity [4]. Loss of skin barrier function induces trans-epidermal water loss (TEWL), alters the skin pH, and causes skin dehydration [5]. The imbalance in Th1/Th2-cytokines, observed in AD, promotes IgE-mediated hypersensitivity and itching. Repetitive itching and scratching are the major symptoms of AD; the itch-scratch cycle leads to loss of sleep and severely disturbs the quality of life of AD patients.

NC/Nga mice have been specifically developed to study AD; these display pathological and behavioral features similar to AD and thus are extensively used as models to study AD [6]. Exposure to 2,4-dinitrochlorobenzene (DNCB) induces stable, clinical AD-like skin lesions in NC/Nga mice that present an allergic inflammatory response and scratching behavior [7,8]. This is followed by rapid development of erythema, lichenification with edema along with elevated levels of total serum IgE [9].

Clinical treatment of AD generally includes topical application of steroids; corticosteroids are highly effective in reducing inflammation and pruritus, and bacterial colonization of the skin, thus strengthening the skin barrier [10]. However, frequent and long-term use of topical steroids is associated with various adverse effects, including skin atrophy, telangiectasia, and hypertrichosis [11]. Moreover, “steroid phobia” makes patients unwilling to take steroids [12]. Given the negative effects of long-term use of topical and systemic steroids, many studies are now focusing on finding potential and effective topical natural agents for treating patients with AD.

The Rural Development Administration of Korea developed resveratrol-enriched rice (RR), a transgenic variety of rice exhibiting synergistic effects of resveratrol (RSV) and rice. Resveratrol is a natural polyphenol found in various types of fruits and vegetables, mainly red grapes and berries. It is reported to exert anticancer, antioxidant, antiangiogenic, and anti-inflammatory effects [13]. Resveratrol has been reported to effectively decrease inflammation and histological changes by modulating apoptosis and regulating the secretion of cytokines in the epithelium in a DNFB-induced murine model [14]. On the other hand, rice (*Oryza sativa* var. *japonica*) is consumed by Asians in large quantities. Moreover, it is widely used by the cosmetic industry as part of skin-lightening products and to prevent aging and wrinkle formation [15]. Clinically, various rice components are known to improve skin barrier function by preventing epidermal water loss by regulating levels of lipids, such as glucosylceramides and ceramides [16]. Given these studies on biological activity of RSV and rice, RR might have synergistic effects stronger than resveratrol or rice alone. The grains of RR contained as much resveratrol as high-quality red wine and long-term RR supplementation has anti-obesity effects, such as reducing body weight and abdominal fat volume, without causing serious side effects [17]. It also shows an anti-aging effect by attenuating UVB-ROS induced skin inflammation [18] and an anti-melanogenic effect by downregulating tyrosinase and tyrosinase-related protein (TRP-2) expression [19]. Genetically engineered RR might be a rich natural source of biologically active RSV, manifesting the synergistic anti-inflammatory effect of RSV and normal rice. Therefore, we examined the inhibitory effect of RR on AD-like skin lesions in in vitro and in vivo.

## 2. Results

### 2.1. Resveratrol-Enriched Rice Suppressed AD-like Skin Severity in DNCB-Induced NC/Nga Mice

**Figure 1:**
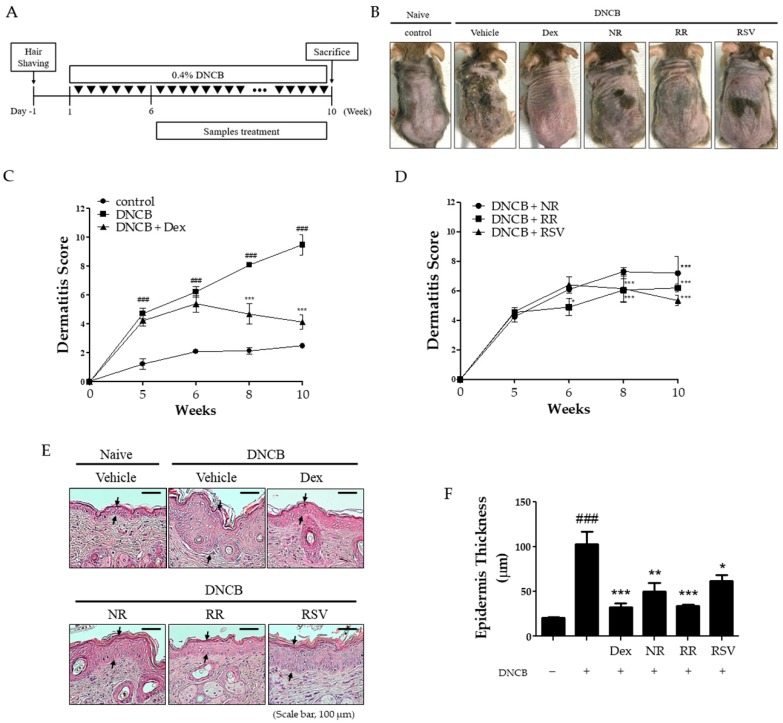
Effect of RR on the development of atopic dermatitis (AD) in DNCB-induced NC/Nga mice. (**A**) Schematic presentation of the animal experiment schedule. Mice were divided into five groups (*n* = 7 per group). After the induction of AD-like symptoms with DNCB, NR, RR, and RSV were topically applied thrice weekly for five weeks. (**B**) Images of dorsal skin were taken on the last day of the experiment. (**C**,**D**) The dermatitis score was defined as the sum of score for five clinical indexes: erythema, edema, erosion, dryness, and lichenification. (**E**) Hematoxylin and eosin staining of dorsal skin. Scale bar = 100 μm. Arrows indicate the thickness of epidermis. (**F**) Histogram of hematoxylin and eosin staining. Values are expressed as means ± SEM. ^###^ *p* < 0.001 versus vehicle group; * *p* < 0.05, ** *p* < 0.01, and *** *p* < 0.001 versus DNCB-treated group. Dex, dexamethasone; NR, normal rice; RR, resveratrol-enriched rice; RSV, resveratrol.

To assess the effect of RR against the development of AD-like skin lesions, NC/Nga mice were treated with Dexamethasone (Dex 0.1% (*w*/*v*)), normal rice extract (NR, 2.5% (*w*/*v*)), RR rice extract (RR, 2.5% (*w*/*v*)), and RSV (2.5% (*w*/*v*)) thrice weekly for five weeks after sensitization with 0.4% (*w*/*v*) DNCB (Figure 1A). As shown in Figure 1B–D, repeated application of DNCB resulted in AD symptoms, such as erythema, scaliness, dryness, thickening, and lichenification as compared with the vehicle group. Furthermore, H&E staining demonstrated thickening of the epidermis in the DNCB-treated group compared with the vehicle group (Figure 1E). The NR, RR, and RSV treatments appeared equally capable of reducing AD severity, but RR treatment showed more recovery effect than NR treated group. RR also reduced epidermis thickening more effectively and significantly than RSV or NR treatment alone (Figure 1F). These results demonstrated RR treatment to decrease AD symptoms in DNCB-induced NC/Nga mice.

### 2.2. Resveratrol-Enriched Rice Reduced Scratching Behavior, Serum IgE, and IL-31 Expression in DNCB-Induced NC/Nga Mice

**Figure 2:**
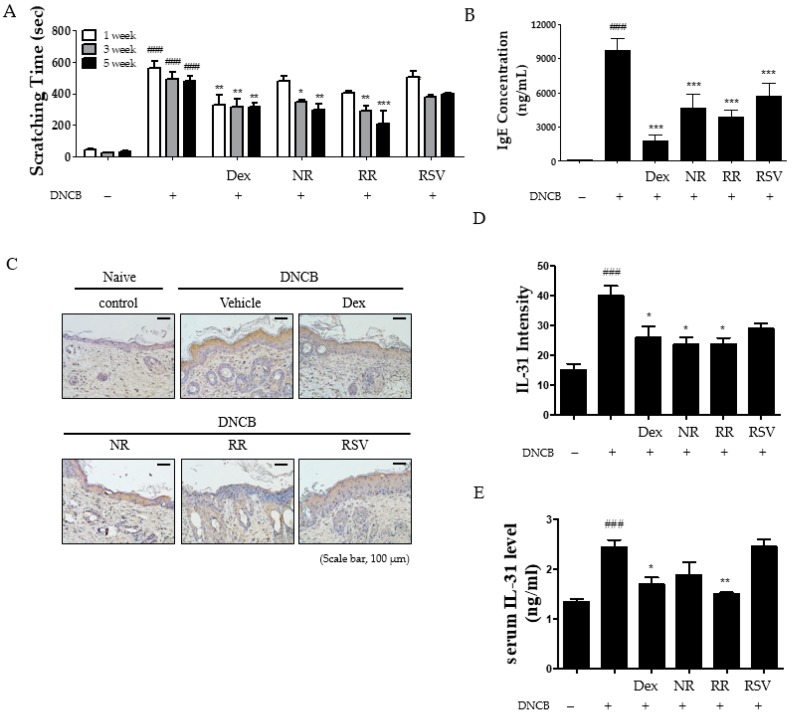
Effect of RR on pruritus in DNCB-induced NC/Nga mice. (**A**) Scratching frequency was measured via videotaping mice from above for 20 min. (**B**) The level of serum IgE was measured using ELISA. (**C**) Expression of IL-31 in DNCB-induced NC/Nga mice was measured using IHC. Scale bar, 100 μm. (**D**) Graphical representation of expression of IL-31 in dorsal skin. (**E**) The level of serum IL-31 was measured using ELISA. Values are expressed as means ± SEM. ^###^ *p* < 0.001 versus vehicle group; * *p* < 0.05, ** *p* < 0.01, and *** *p* < 0.001 versus DNCB-treated group.

To investigate antipruritic effects of RR on DNCB-induced NC/Nga mice, we monitored and calculated the time that mice spent scratching their dorsal skin with their hind paws. We observed the duration of scratch behavior to be consistently longer in DNCB-induced mice than in vehicle-treated controls (Figure 2A). The vehicle-treated group exhibited scratching for less than 1 min within a window of 20 min in all weeks, whereas the DNCB-treated group scratched for approximately 10 min during this time span. The RR-treated mice exhibited a significantly reduced scratching frequency compared with Dex- and NR-treated groups at the five-week time point. We measured the levels of serum IgE, an important inflammatory mediator in AD, to gain additional evidence for the antipruritic effects. The IgE levels in the plasma of DNCB-treated mice were found to be considerably higher (increased to the 1-μg scale) than the vehicle group. Treatment with RR and Dex significantly reduced the IgE levels by approximately 80% and 50%, respectively (Figure 2B). Furthermore, we measured the expression of IL-31, a pruritogenic cytokine in AD, in the dorsal skin using immunohistochemistry (IHC), which reported an increase of approximately twofold by repeated DNCB application as compared with the vehicle group (Figure 2C). Both RR and Dex treatments reduced IL-31 expression to a similar level (Figure 2D). Furthermore, ELISA assay showed that RR also reduced IL-31 in serum significantly. These data suggest the ability of RR treatment to suppress the development of AD-like skin lesions by inhibiting itching through regulation of serum IgE level and IL-31 expression.

### 2.3. Resveratrol-Enriched Rice Ameliorates TEWL and Skin Hydration in DNCB-Induced NC/Nga Mice

**Figure 3:**
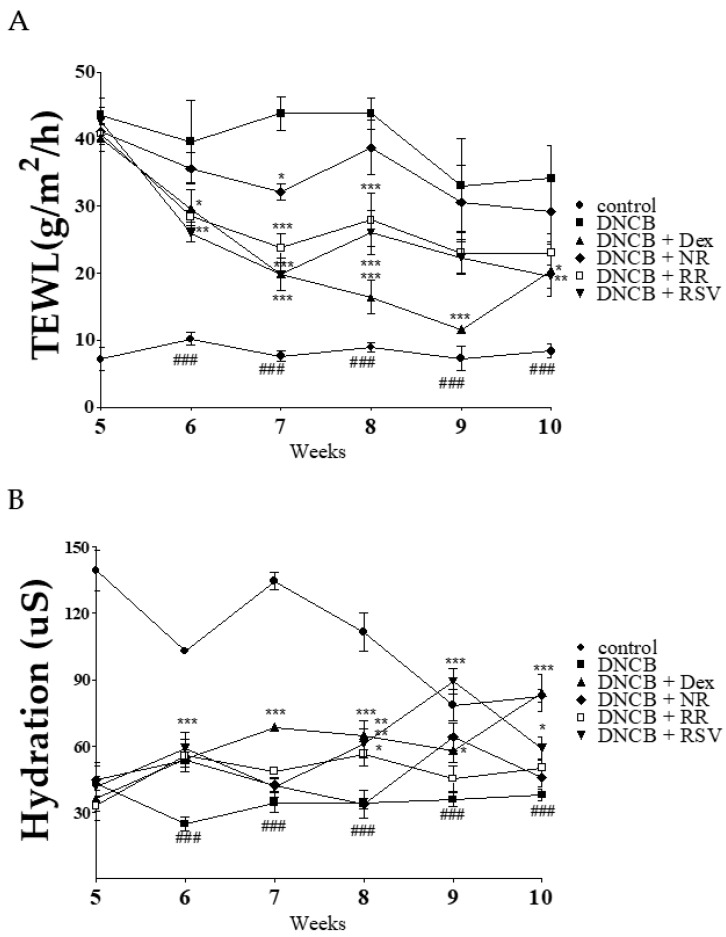
Effect of RR on TEWL and hydration in DNCB-induced NC/Nga mice. (**A**) The level of TEWL; and (**B**) the level of hydration in NC/Nga mice dorsal skin were measured using the Derma Combo system every week. Values are expressed as means ± SEM. ^###^ *p* < 0.001 versus vehicle group; * *p* < 0.05, ** *p* < 0.01, and *** *p* < 0.001 versus DNCB-treated group. DNCB, 2,4-dinitrochlorobenzene; RR, resveratrol-enriched rice; SEM, standard error of the mean; TEWL, trans-epidermal water loss.

We next examined the effect of RR on skin barrier function in DNCB-induced NC/Nga mice. We measured TEWL and the level of skin hydration every week after DNCB induction. The DNCB-treated group displayed an increase in TEWL score. Hydration level in DNCB-treated group also decreased as compared to vehicle group. TEWL did not change over time in the skin of vehicle group, and skin hydration gradually decreased because NC/Nga mice spontaneously developed AD-like skin (Figure 3A,B). Treatment with RR or RSV significantly reduced TEWL compared to the group treated with Dex, with a slight recovery of skin hydration in DNCB-treated NC/Nga mice.

### 2.4. Resveratrol-Enriched Rice Suppressed TNF-α/INF-γ-Increased Inflammatory Cytokine Secretion in HaCaT Cells and 3D Skin Model

**Figure 4:**
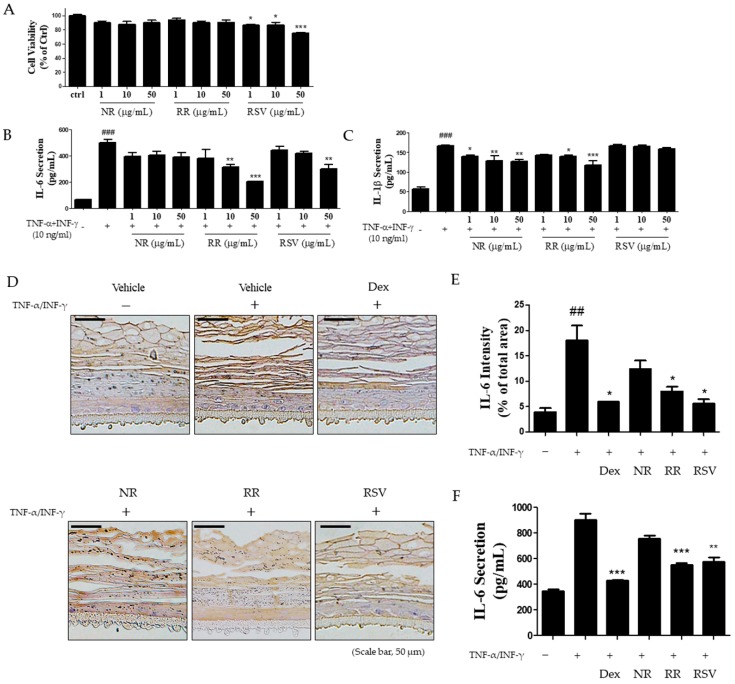
Inhibitory effects of RR on inflammatory cytokine expression in TNF-α/INF-γ-induced HaCaT cells and 3D skin tissue. (**A**) Cells were cultured in 96-well plates and treated with NR, RR, and RSV (1, 10, and 50 μg/mL, respectively). After 24 h, cell viability was measured using the MTT assay. (**B**, **C**) Cells were treated with NR, RR, and RSV in the presence of TNF-α/IFN-γ (each 10 ng/mL). After 24 h, IL-6 and IL-1β levels in supernatants were determined by ELISA. (**D**) The expression of IL-6 in TNF-α/IFN-γ-induced 3D skin model was evaluated by IHC. Vehicle (polyethylene glycol: PBS, 1:1), Dex (1%), NR (1%), RR (1%), and RSV (1%) were applied after TNF-α/IFN-γ treatment in 3D skin model thrice weekly. (**E**) Graphical representation of IL-6 expression in epidermal tissue. Scale bar, 50 μm. (**F**) The level of IL-6 secretion in media was measured using ELISA. Values are expressed as means ± SEM. ^###^ *p* < 0.001 versus control group; * *p* < 0.05, ** *p* < 0.01, and *** *p* < 0.001 versus TNF-α/IFN-γ-treated group.

To investigate an optimal effective range of concentration of RR in human keratinocytes, we treated HaCaT cells with NR, RR, and RSV concentrations ranging from 1 to 50 μg/mL for 24 h. As shown in Figure 4A, no significant changes in cell viability were noticed after RR treatment at these concentrations. We further examined the inhibitory effect of RR on inflammatory cytokine secretion in TNF-α/IFN-γ-stimulated HaCaT cells. RR treatments could significantly inhibit both IL-6 and IL-1β secretions in a dose-dependent manner as compared to NR or RSV treatment alone (Figure 4B,C). We also measured the expression of IL-6 by IHC in the 3D skin tissues: IL-6 was highly expressed in epidermis layer of skin tissue following treatment with TNF-α/IFN-γ. However, RR treatments significantly decreased the level of cytokine expression in skin tissue and secretion (Figure 4D–F). Furthermore, RR treatments in skin of DNCB-induced mice showed that serum level of IL-6 and INF-γ were decreased by RR treatment similar to dexamethasone, but NR and RSV did not significantly reduce INF-γ level (Figure S1). These results imply that RR inhibited TNF-α/IFN-γ-increased secretion of inflammatory cytokines in human keratinocytes.

## 3. Discussion

Atopic dermatitis or eczema is primarily characterized by pruritus. There are mediators capable of stimulating itching, including biogenic amines, proteases, cytokines, and peptides [20]. Various study groups report that the itch–scratch cycle triggers the skin barrier dysfunction, thereby predisposing the epidermis to further foreign environmental and microbial attacks, culminating in skin inflammation. The transmission of itch along both histaminergic and non-histaminergic pathways involves a complex interplay between keratinocytes, immune cells, and cutaneous neurons [21]. Several studies have reported and discussed the antipruritic effects of treatments; however, novel therapeutics targeting the mediators have not yet been investigated [22,23]. In the current study, we observed RR to exhibit distinguished inhibitory effects on both scratching and skin inflammation in AD. It also displayed inhibitory effects on IL-31 expression in the itching skin area. Recently, IL-31 has been reported as a new member of the IL-6 family of cytokines expressed by Th2 cells [24] and is known to be involved in the pathogenesis of such allergic disorders [25]. It mediates its effects through an IL-31 receptor A (IL-31RA), a heterodimeric receptor, and the oncostatin M receptor (OSMR), which is expressed on monocytes, dendritic cells, and keratinocytes [26]. One study reports significantly higher levels of IL-31 in patients with AD than in normal individuals [27], whereas another study reports increased expression of IL-31 receptor in atopic-like skin lesions in an animal model [28]. IL-31 has been known to act as a strong pruritus-inducing agent; however, the underlying mechanism remains unclear [29]. This is evident by the elevated levels of IL-31 and IgE associated with chronic excoriation, thereby resulting in the exacerbation of itch [30]. In addition, Th1- and Th2-associated cytokines, namely INF-γ and IL-4, which are secreted from activated skin-infiltrating T cells, and IL-31, together enhance chemokine production in keratinocytes via phosphorylation of STAT-3 [31]. It is proposed that IL-31 disrupts the protective skin barrier function by weakening the formation of lipid envelope and downregulating the expression of filaggrin [32]. Along these lines, in the present study, we observed high expression of IL-31 in dorsal skin area in DNCB-induced mice that might be related to high frequency of scratching behavior and skin barrier dysfunction. Application of RR suppressed the scratching behavior frequency in AD-like lesions in mice in the early phase and chronic itch in the late phase as compared to treatment with RSV or NR alone. Moreover, RR treatment reduced IL-31 production compared to Dex in the epidermis.

Another important process in the pathogenesis of AD is IgE hyperactivity; its high serum levels mediate the critical features of the AD by binding to mast cells leading to the release of inflammatory mediators. We found increased production of IgE in DNCB-induced mice, and RR treatments led to a notable reduction in its serum levels. Thus, our data suggest that the suppressive effects of RR on itching and skin dysfunction might be mediated, in part, by inhibiting IgE and IL-31 expression.

The epidermis serves to act as a physical barrier against dangerous environmental agents. It is believed that a defective permeability barrier predisposes the skin to external agents and allergens, promoting their entry. Furthermore, a scratch in the epidermal layer can serve as a breach, allowing easy access of allergens to immune system [33]. One of the consequences of a defective permeability barrier is elevated TEWL, a marker for skin barrier function, owing to damaged stratum corneum and corneocyte adhesion loss [34]. We noticed an increased TEWL and skin dehydration in NC/Nga mice following DNCB-induced AD. As expected, NR, RR, RSV, and Dex treatments gradually reduced elevated TEWL after induction of DNCB and RR treatment showed skin damage recovery compared to RSV treatment not because of NR treatment. Furthermore, DNCB-treated group displayed the lowest skin hydration level throughout the experiment. However, RR could not increase the skin hydration level significantly after DNCB induction even at the end of the experiment, whereas RSV could recover stratum corneum water content. These findings suggest the ability of RR to maintain and recover epidermal skin barrier function and modulate AD severity, similar to RSV treatment.

Another manifestation of AD is the alteration of the inflammatory molecules of the immune system. Keratinocytes in patients with AD exhibit an increased production of cytokines and chemokines. This is corroborated by the finding that stimulation of keratinocytes with Th2-mediated cytokines triggers inflammatory response [35], highlighting the regulation of cytokine production in keratinocytes as another key player in the pathophysiology of AD. We studied the synergistic effects of RR on keratinocytes and demonstrated that RR potently suppressed skin inflammation by inhibiting expression of pro-inflammatory cytokines, such as IL-6 and IL-1β in TNF-α/INF-γ-induced human keratinocytes and 3D human skin tissue model. Previously, Subedi et al. observed that the anti-aging effect of RR against UVB-induced photoaging in NHDF cells in vitro [18]. Thus, RR treatment not only inhibited the oxidative stress induced by UVB but also significantly inhibited the inflammation cascades. As RR did not show any significant toxicity to NHDF or HaCaT cells, we expect that it might not show any toxicity to human skin as well. Also, we checked that RR contained abundant amount of resveratrol (Figure. S2). Our results conclude that RR exerts an effective anti-inflammatory effect through regulation of IL-6 and IL-31 cytokines, which in turn reduce the thickening of epidermis, activation of mast cells-producing IgE, skin erythema, dryness, edema, and erosion. Even though RR treatment more effectively reduced skin dryness and recovered TEWL than the NR treatment, the anti-inflammatory effect of RR is almost the same as NR, thus further study will be conducted to address the beneficial effect of RR.

## 4. Materials and Methods

### 4.1. Preparation of Rice Extracts

RR was developed by the Rural Development Administration of Korea using genetic engineering. RR powder and normal rice (NR) powder (50 g) were soaked in 80% methanol (500 mL) for 24 h with 1 h of sonication. The solution was filtered through Whatman No. 1 filter paper and concentrated using a rotary vacuum evaporator, freeze-dried, and stored at −80 °C until use. The extract was dissolved in an acetone for in vivo or dimethyl sulfoxide (DMSO) for in vitro use

### 4.2. Animal Experiments

Specific pathogen-free, 5-week-old NC/Nga mice were obtained from Orient Bio Inc. (Gyeonggi-do, Korea). Prior to experiments, all mice were acclimated for 1 week to the following conditions: temperature of 23 °C, 65% humidity, and 12/12 h light/dark cycle. Animals were provided with food and water ad libitum. All experimental procedures utilizing animals were reviewed and approved by the animal care committee of the Center of Animal Care and Use (CACU; LCDI-2016-0027, 22 March 2016) at the Lee Gil Ya Cancer and Diabetes Institute, Gachon University, Korea. To induce AD-like skin lesions, hairs from approximately 8 cm² of the dorsal skin of mice were removed, following which 200 µL of 0.4% DNCB solution (dissolved in a 1:3 mixtures of acetone and olive oil) was applied twice per week for five weeks. Mice were divided at random into five treatment groups (7 mice per group): (1) naive mice (vehicle); (2) DNCB only (DNCB 0.4% (*w*/*v*)); (3) DNCB + Dexamethasone (Dex; 0.1% (*w*/*v*), 0.5 mg/200 uL); (4) DNCB + Normal Rice (NR; 2.5% (*w*/*v*), 5 mg/200 uL); (5) DNCB + Resveratrol enriched rice (RR; 2.5% (*w*/*v*), 5 mg/200 uL); and (6) DNCB + Resveratrol (RSV 2.5% (*w*/*v*), 5 mg/200 uL). After induction of AD, RR was topically applied to the dorsal skin and ears twice per week for five weeks (Figure 1A). In RR-treated mice, 200 μL of RR solution was applied topically 1 h before DNCB application each time. The severity of dermatitis was assessed once a week by three persons, according to the method described by Leung [36]. The total clinical index of dermatitis severity was defined as the sum of individual scores graded as follows: 0 (none), 1 (mild), 2 (moderate), and 3 (severe) for each of the five signs and symptoms: erythema/hemorrhage, edema/hematoma, excoriation/erosion, itching/dryness, and lichenification.

### 4.3. Scratching Behavior

The scratching frequency was recorded using digital camera (Coolpix A300, Nikon imaging Korea, Seoul, Korea) and calculated the time of NC/Nga mice spent scratching their dorsal skin using their hind paws for 20 min. Animals were sacrificed 10 weeks after the first application of DNCB for collecting skin tissue and blood.

### 4.4. TEWL and Skin Hydration

TEWL in mouse dorsal skin was measured under specific conditions of 21–22 °C and 50–55% humidity using a Dermalab Combo system (C40000.03-189, Cortex Technology, Hadsund, Denmark) once a week. The measurements were recorded when TEWL readings stabilized at approximately 30 s after the probe was placed on the skin. The data were analyzed with a microprocessor and expressed in g/m²/h. Hydration of the stratum corneum was also measured using a Dermalab Combo system. The instrument is based on the measurement of high-frequency electrical conductance. Three independent measurements of the same skin area were averaged for each value.

### 4.5. Cell Culture and 3D Skin Model

The spontaneously immortalized human keratinocyte cell line HaCaT was obtained from the Korean Cell Line Bank (Seoul, Korea). Cells were cultured in DMEM supplemented with 10% fetal bovine serum (FBS) and 100 unit/mL penicillin and 100 μg/mL streptomycin. Cells were then incubated in a humidified atmosphere of 5% CO_2_ at 37 °C. Cells were treated with RR for 1 h and stimulated with or without TNF-α and IFN-γ (each 10 ng/mL) for 24 h in serum-free culture medium. The KeraSkin™ model (Biosolution, Seoul, Korea) was transferred onto a 6-well plate with 1 mL of KeraSkin growth medium and incubated overnight at 37 °C with 5% CO_2_. To induce skin inflammation, TNF-α or IFN-γ (10 ng/mL), dissolved in a solution of polyethylene glycol and PBS (1:1), was applied twice a week before sample treatment. TNF-α or IFN-γ was removed with repeated rinsing with PBS, and Dex (1% (*w*/*v*)), NR (1% (*w*/*v*)), RR (1% (*w*/*v*)), and RSV (1% (*w*/*v*)) were applied thrice weekly for two weeks.

### 4.6. Cell Viability

The MTT assay was used to measure the cytotoxicity of RR. For this, HaCaT cells were seeded into a 48-well plate (6.0 × 10^4^ cells/well) in 10% FBS-containing medium. After 24 h incubation, 0.5 mg/mL MTT solution (Sigma-Aldrich, St. Louis, MO, USA) was added to the wells and cells were continuously cultured for 1 h. The dark blue formazan crystals were solubilized with DMSO, and the absorbance was measured at 570 nm using a spectrophotometer (Molecular Devices; San Jose, CA, USA).

### 4.7. ELISA

IL-6 and IL-1β secretions were measured using an enzyme-linked immunosorbent assay (ELISA). HaCaT cells were seeded (3 × 10^5^ cells/well) in a 24 well plate and stimulated with 10 ng/mL TNFα/INFγ in the presence of NR, RR and RSV. After 24 h, the supernatants were collected and IL-6 and IL-1β secretions were evaluated using their respective ELISA kit (R&D Systems, Minneapolis, MN, USA). For measurement of serum total IgE level and IL-31, blood was collected from the mice on the last day of sacrifice. Serums were obtained by centrifugation (12,000 rpm, 10 min) and stored at −80 °C. Total serum IgE quantification was performed using a mouse IgE measurement kit (Invitrogen, Carlsbad, CA, USA) and IL-31 quantification was performed using IL-31 ELISA kit (R&D Systems, Minneapolis, MN, USA).

### 4.8. Histology and Immunohistochemistry

The 3D skin model and animal skin tissues were fixed with 10% buffered-neural formalin. The fixed tissues were embedded into paraffin wax and sectioned into 5-μm-thick sections. The sections were then stained using hematoxylin and eosin (H&E) and visualized under the microscope (Olympus; Tokyo, Japan) for histological analysis. ImageJ software was used to analyze the images. For each mouse, the epidermal length was measured in ten randomly-selected fields of view. To monitor the expression of cytokines, 4-μm sections of tissue were prepared. Antigen retrieval was performed using 20 μg/mL proteinase (in PBS) for 20 min at 37 °C. The sections were then incubated with 3% H_2_O_2_ in PBS for 15 min to quench the endogenous peroxidase activity. The sections were incubated with anti-IL-31 antibody (abcam, diluted 1:100) for 12 h at 4 °C. After incubation, sections were washed with PBS to remove the excess primary antibody and incubated with the secondary antibody (1:200) for 20 min at room temperature. After being rinsed twice with PBS, the sections were incubated in VECTASTAIN ABC reagent (Vector Laboratory; Piscataway, NJ, USA) for 30 min. Immunoreactions were visualized using 3,3′-diaminobenzidine (DAB, Vector Laboratory; Piscataway, NJ, USA). The sections were counterstained with hematoxylin for 3 min and visualized under the light microscope (Olympus; Tokyo, Japan).

### 4.9. Statistical Analysis

Results of statistical analyses were expressed as mean ± SEM. Statistical comparisons were conducted between control and various groups using Bonferroni’s test for multiple comparisons of one-way analysis of variance (ANOVA) using GraphPad Prism 5.0 (GraphPad Software Inc., San Diego, CA, USA). A *p*-value less than 0.05 was considered statistically significant.

## 5. Conclusions

The results of the present study suggest that topical application of NR, RR, and RSV attenuated the skin barrier dysfunction and pruritus in DNCB-treated NC/Nga mice with a concomitant decrease in the expression of epithelial-derived cytokines and thus reduced cytotoxicity. However, among these, RR exhibited the most potent anti-inflammatory and skin repair activities, and antipruritic effects, a manifestation of the synergistic effect of RSV and NR. Hence, as compared to Dex, which is widely used in therapy of AD, RR is indicated to possess advantages of both RSV and NR, with low side-effects. In light of these findings, we suggest RR as a potential and alternative therapy to treat pruritic and inflammatory effects of AD. However, further studies are warranted to reveal the mechanism of action of RR at the molecular level.

## Supplementary Materials

The following are available online at https://www.mdpi.com/1422-0067/20/6/1428/s1.
