# Supplementary material for: Effect of Resveratrol-Enriched Rice on Skin Inflammation and Pruritus in the NC/Nga Mouse Model of Atopic Dermatitis
Source: Int J Mol Sci. 2019 Mar 21;20(6):1428. doi: 10.3390/ijms20061428 (PMC6471349; doi:10.3390/ijms20061428)
Supplement: Supplementary file 1 [file ijms-20-01428-s001.pdf]

International Journal of *Molecular Sciences*

Supplementary:

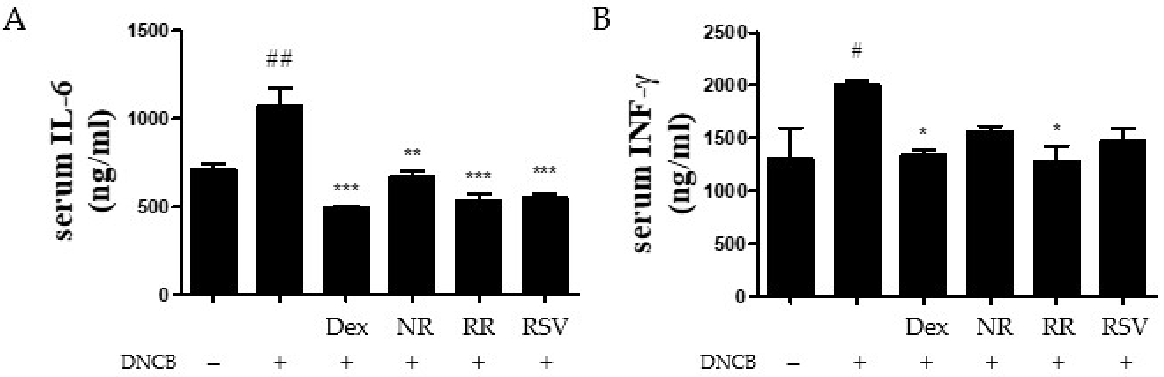

Figure 1. Inhibitory effects of RR on inflammatory cytokine expression in serum of DNCB-induced NC/Nga mice. The level of serum IL-6 (**A**) and INF-gamma (**B**) were measured using ELISA.

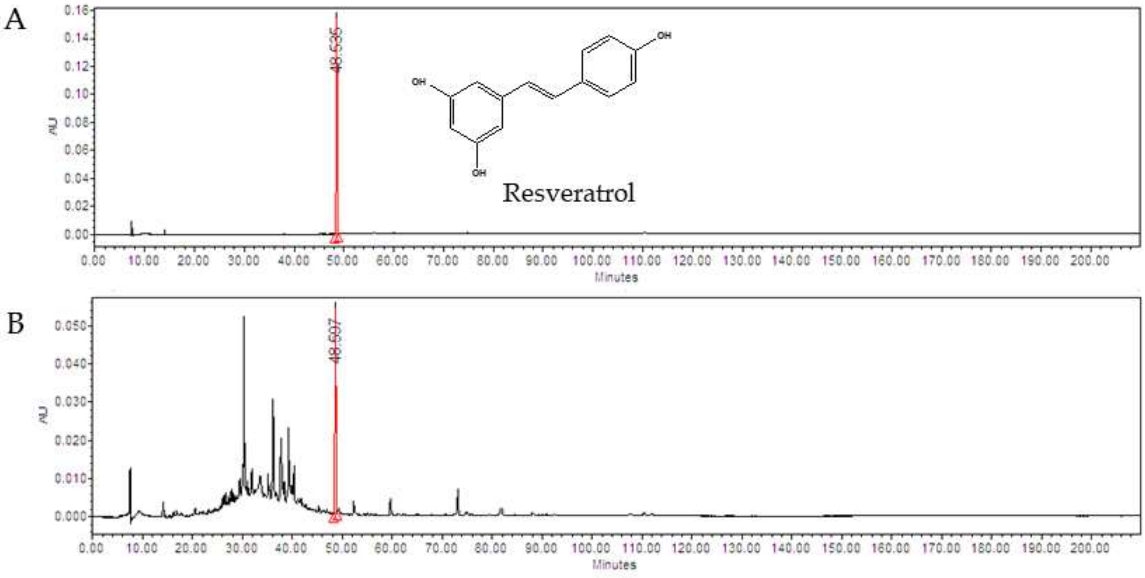

Content of resveratrol in RR extract (μg/1000 mg of powdered) at 306 nm.	
**Sample**	**Resveratrol**
RR extract	3.59 ± 0.65

Figure 2. Contents of Resveratrol in the RR extract.
